# Data on investigating the nitrate concentration levels and quality of bottled water in Torbat-e Heydarieh, Khorasan razavi province, Iran

**DOI:** 10.1016/j.dib.2018.08.031

**Published:** 2018-08-14

**Authors:** Hamed Akbari, Hamed Soleimani, Majid Radfard, Abbas Abasnia, Bayram Hashemzadeh, Hesam Akbari, Amir Adibzadeh

**Affiliations:** a Health Research Center, Lifestyle institute, Baqiyatallah University of Medical Sciences, Tehran, Iran; b Department of Environmental Health, School of public Health, Tehran University of Medical Sciences, Tehran, Iran; c Khoy University of Medical Sciences, Khoy, Iran

**Keywords:** Bottled water, Nitrate, Microbial quality, Torbat-e Heydarieh, Iran

## Abstract

The human body is primarily water and healthy drinking water is vital to human life. Today, the bottled-water industry has been widely developed in most countries and more than 150 several brands of bottled water are produced in Iran. Considering the increasing consumption of bottled water and its potential for contamination with harmful chemical and microbial agents such as nitrate, the aim of this study was to assess the nitrate concentration and also the microbial quality of bottled water in a number of brands produced in the Torbat-e Heydarieh city in 2017. In present descriptive-analytical research, random sampling (80 samples) was done by collecting 1.5 l bottled water with different production dates from 20 factories. These samples were collected in four different seasons. Measurement of nitrate concentration and microbial quality including total and fecal coliforms, were performed according to the Standard Methods for the Examination of Water and Wastewater. The results indicated that, in general, the mean concentration of nitrate in all samples was range 0.6–16 mg/L and all samples are within the national standard of Iran (less than 50 mg/L) and international standards. Also, total coliforms and fecal coliforms in any of the studied samples were zero.

**Specifications Table**

**Table:** 

Subject area	Water chemistry and microbiology
More specific subject area	Water nitrate
Type of data	Table, Figure
How data was acquired	The nitrate concentration was measured by spectrophotometer Hach (DR 5000 model) and in 220 nm wave length. Microbial parameters measurements, including the most probable number of coliforms (MPN) and fecal coliforms by multi-pipe fermentation method [1], [2], [3], [4], [5], [6], [7], [8], [9], [10].
Data format	Raw, analyzed
Experimental factors	The mentioned parameters above, in abstract section, were analyzed according to the standards for water and wastewater treatment handbook.
Experimental features	Very brief experimental description
Data source location	Torbat-e Heydarieh, Khorasan-e-Razavi province, Iran
Data accessibility	The data are available with this article

**Value of the data**

• Nitrate and nitrite compounds are among the contaminating factors of groundwater resources.
• Assurance that water is microbiologically safe for drinking has traditionally been determined by measuring bacterial indicators of water quality, most commonly, total coliforms and fecal coliforms.
• Data presented in this article showed that the nitrate concentration and the microbial quality of bottled water in Torbat Heydarieh were within the standard levels, therefore, it does not pose a risk to the health of consumers.

## 1 Data

**Fig. 1:**
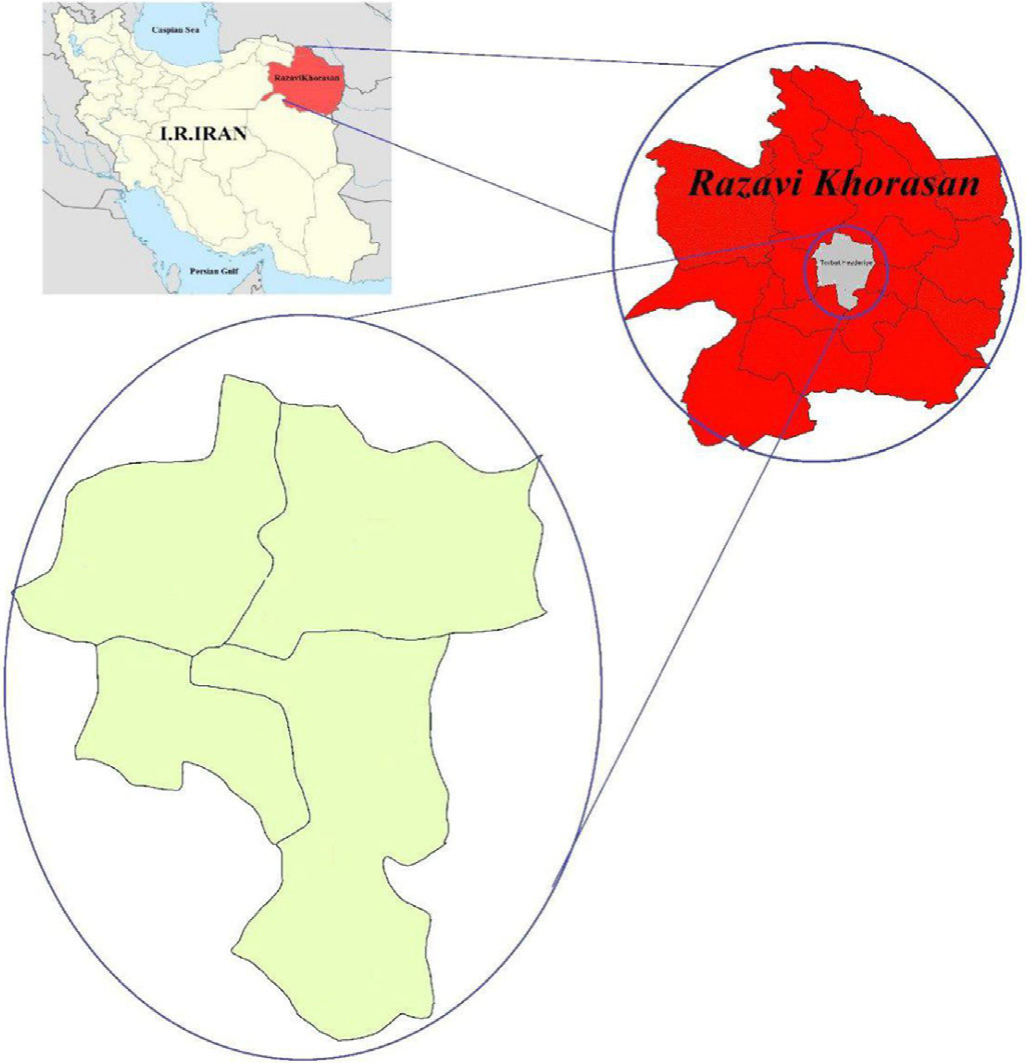
Study area.

Data presented here deal with monitoring of nitrate concentration and microbial quality of bottled-water in Torbat-e Heydarieh city, Khorasan-e-Razavi province, Iran. Fig. 1 shows location of study area. Also, Table 1 shows average of nitrate concentration of bottled-water in Torbat-e Heydarieh city.

**Table 1:** Nitrate concentration and microbial parameters of bottled-water in Torbat-e Heydarieh city.

**Bottled water samples**	**Mean of nitrate concentration**	**Microbial parameters**
	**measured in samples**	**coliforms (MPN) and fecal coliforms**
B1	12.23 ± 1.22	0
B2	9.31 ± 0.92	0
B3	8.88 ± 1.50	0
B4	6.26 ± 1.07	0
B5	16.64 ± 2.45	0
B6	5.26 ± 0.86	0
B7	2.23 ± 0.64	0
B8	9.22 ± 0.93	0
B9	13.90 ± 2.12	0
B10	7.44 ± 1.03	0
B11	3.66 ± 0.79	0
B12	8.34 ± 1.06	0
B13	13.90 ± 3.13	0
B14	10.33 ± 2.42	0
B15	9.12 ± 2.17	0
B16	7.80 ± 1.08	0
B17	6.50 ± 0.89	0
B18	8.90 ± 1.07	0
B19	6.66 ± 0.93	0
B20	7.50 ± 1.05	0
Mean	8.7	0

## 2 Experimental design, materials and methods

### 2.1 Study area description

Torbat-e Heydarieh is a city and capital of Torbat-e Heydarieh County, in Khorasan Razavi Province, Iran. At the 2016 census, its population was 140,019. Torbat Heydarieh is located between latitudes 35°.2798´ N and longitudes 59°.2161´ E, encompassing an area of about 3900 km^2^ and the average altitude of the city is 1333 m above sea level [1].

### 2.2 Sample collection and analytical procedures

This descriptive-analytical research was done by examining nitrate concentrations and microbial quality of bottled water in Torbat-e Heydarieh city in 2017. Experiments were performed on eighty samples of 1.5 l bottled water, with different production dates. These samples were collected from 20 best-selling and most popular brands of bottled water produced from different factories, in four stages (in four seasons of the year). Random sampling was done by purchasing water from supermarkets. The nitrate concentration was measured by spectrophotometer Hach (DR 5000 model) and in 220 nm wave length [11], [12], [13], [14], [15], [16], [17], [18]. Microbial parameters measurements including the most probable number of coliforms (MPN) and fecal coliforms by multi-pipe fermentation method were performed on collected samples and compared with the national standard of Iran and also, the WHO and EPA guidelines [19], [20], [21], [22], [23], [24]. In the end, descriptive statistics (The Mean and Standard deviation) were used to summarize the data of the tests.
